# Impact of enhanced recovery after surgery (ERAS) on postoperative morbidity and long-term survival in patients with perihilar cholangiocarcinoma

**DOI:** 10.1007/s00423-026-04100-8

**Published:** 2026-06-10

**Authors:** Robert Oehring, Eriselda Keshi, Marlen Breitkreuz, Alexandra Zuehlke, Sebastian Knitter, Simon Moosburner, Pia F. Koch, Wenzel Schöning, Johann Pratschke, Jens Neudecker, Felix Krenzien

**Affiliations:** 1 Department of Surgery, Campus Charité Mitte and Campus Virchow-Klinikum, Charité- Universitätsmedizin, corporate member of Freie Universität Berlin, Humboldt-Universität zu Berlin, Augustenburger Platz 1, Berlin, 13353 Germany; 2 Berlin Institute of Health (BIH), Berlin, Germany; 3 Department of Surgery, Harzklinikum D.C. Erxleben, Quedlinburg, Germany

**Keywords:** Enhanced Recovery after Surgery, Perihilar cholangiocarcinoma, Klatskin, Complications

## Abstract

**Purpose:**

Enhanced Recovery After Surgery (ERAS) is a multimodal perioperative care pathway designed to improve surgical outcomes and has been widely adopted across surgical disciplines. This study evaluated the impact of adherence to ERAS Society recommendations on postoperative complications and survival in patients undergoing surgery for perihilar cholangiocarcinoma (PCH).

**Methods:**

A total of 168 patients with PCH who underwent extended liver surgery with hepatobiliary reconstruction between 2014 and 2024 were included. Of these, 94 patients were treated according to the official ERAS protocol, while 74 patients treated prior to ERAS program implementation served as the control group (Non-ERAS). The ERAS program followed strictly the official ERAS Society guidelines. Patients were analyzed with regard to postoperative complications, adherence to ERAS measures, and long-term survival.

**Results:**

ERAS was associated with a significant reduction in overall complications (Clavien-Dindo grades 1–5), Non ERAS 97.3% vs. ERAS 83.0% (*p* = 0.003). Within the ERAS group, lower complication rates were observed in patients with higher adherence (≥ 50%) compared to those with lower adherence (< 50%), 78.2% vs. 100% (*p* = 0.031). Overall adherence in the ERAS group was 64.3%, with the highest adherence seen for preoperative items (80.7%). When ERAS was coordinated by dedicated ERAS staff, overall survival was significantly improved compared to the non-ERAS group (*p* = 0.037). However, this association was no longer observed after multivariable adjustment. Instead, the administration of adjuvant chemotherapy and younger age emerged as independent predictors of prolonged survival.

**Conclusions:**

Implementation of the ERAS program in patients undergoing extended liver resection for PCH is feasible and safe. It was associated with a reduction in postoperative complications and a trend towards improved overall survival. These effects were most pronounced when the ERAS protocol was guided and supervised by dedicated ERAS staff.

## Introduction

Perihilar cholangiocarcinoma (PHC) is located at the bile duct bifurcation and is the second most common liver cancer. Major hepatectomy is an established potential curative treatment option [1–3]. The 5 year survival rate in R0 resected patients ranges between 11 and 45% [4, 5]. However, extended liver resection with hepatobiliary reconstruction is considered high risk with high complications rate [5–7]. In a benchmark study across 24 high-volume centers (*n* = 1,829), Mueller et al. reported 75th-percentile rates of 87% for any complication and 70% for Clavien–Dindo grade ≥3a [8].

Therefore, improving outcomes remain of superior importance. One cornerstone is perioperative strategies that cover all phases of surgical treatment and related care. The so-called Enhanced Recovery after Surgery (ERAS) is a multimodal approach. A dedicated ERAS protocol for liver surgery consists of different items with different levels of evidence [9, 10]. The guidelines have been recently prospectively validated for liver surgery by our group demonstrating a reduction of complications. Schmelze et al. could show a decrease in overall complications from 41.2% in the Non-ERAS group to 26.5% in the ERAS group *p* = 0.0423, which was mainly attributed due to the reduction of grade 1–2 complications from 17.6% to 7.6%, *p* = 0.0322 [11]. Although earlier studies suggested a reduction in complications, they did not clarify which types were affected (e.g., pneumonia, bile leakage). Oehring et al. addressed this in a larger cohort of 1,049 patients, demonstrating a significant decrease in non-surgical complications mostly due to infectious complications including wound infections and urinary tract infections (non-ERAS 27.6% vs. ERAS 16.3%; *p* = 0.033) [11, 12]. Quinn et al. demonstrated the feasibility of ERAS in PHC in a cohort of 60 patients; 46% completed the protocol with complete datasets. Morbidity remained high, with an overall complication rate of 87% [13]. Their study indicates that ERAS implementation in PHC is possible and especially high risk patients and those with major complications can be appropriately managed in an ERAS pathway. Nevertheless, robust studies evaluating the impact of the official ERAS protocol on complications or survival in PHC are still lacking.

Accordingly, this observational study aimed to evaluate ERAS adherence, postoperative complication rates, and long-term survival in patients with PHC undergoing major liver resection, comparing outcomes before and after ERAS implementation.

## Methods

We identified all consecutive patients who underwent surgery for perihilar cholangiocarcinoma (PHC) at the Department of Surgery, Charité – Universitätsmedizin Berlin (Campus Charité–Mitte and Campus Virchow-Klinikum) between January 2014 and July 2024. Data were retrieved from a prospectively maintained institutional database at the Department of Surgery, Charité – Universitätsmedizin Berlin, Germany (EIAS data base). The analysis was conducted retrospectively. The study was approved by the local ethics committee (EA4/158/25) and performed in accordance with the Declaration of Helsinki and its later amendments. Patients treated before ERAS implementation (2014–2018; *n* = 74) received standard perioperative care and served as the control cohort. After ERAS adoption (2019–2024; *n* = 94), management followed the ERAS Society protocol [9]. Patients were assigned to Non-ERAS and ERAS cohorts. The ERAS cohort was further divided into two groups: (i) ERAS with staff, meaning patients were actively coordinated and closely followed by dedicated ERAS personnel; and (ii) ERAS without staff, meaning patients were treated after ERAS implementation but without dedicated ERAS supervision. The latter resulted from limited ERAS staffing during early implementation, gradual team expansion over time, temporary interruptions during the COVID-19 pandemic, and occasional understaffing around holiday periods such as Christmas and New Year. Even without direct ERAS staff supervision, perioperative care followed the ERAS protocol, which had been institutionalized at our centre. However, several key preoperative elements could not be delivered at all without ERAS staff supervision, including structured patient education, nutritional risk screening, and targeted optimization for undernourished patients (e.g., preoperative enteral supplementation). Intraoperative adherence was largely ensured owing to a high degree of standardization. Postoperatively, ERAS principles were generally applied across wards after comprehensive staff training; however, in the absence of an ERAS nurse, structured visits for four consecutive days, and longer when indicated, could not always be guaranteed.

When interpreting compliance data, it is important to consider that certain ERAS items were frequently not applicable within our institutional setting. For instance, the ERAS Society recommends initiating prehabilitation measures such as smoking cessation or alcohol abstinence 4 to 6 weeks prior to surgery [9]. However, these patients are scheduled immediately and the mean waiting time is 2 weeks; therefore, a 4-week alcohol-abstinence period is technically not feasible. In addition, several items included in the most recent ERAS guidelines—such as postoperative glycemic control and preoperative biliary drainage—were newly introduced in the 2022 update [9].

### ERAS Setup

The ERAS protocol was implemented 2019 and certified in strict accordance with ERAS society recommendations, documented in the ERAS Interactive Audit System (EIAS; Encare, Stockholm, Sweden), and coordinated by a multidisciplinary team of surgeons, anesthesiologists, and ERAS trained nurses [9].

For each patient, a compliance score was calculated based on adherence to 28 ERAS items, as recorded in the system. ERAS staff serve as the central coordinators of the program, ensuring adherence throughout the perioperative pathway. Their core responsibilities include preoperative patient education, coordination of multidisciplinary care, facilitation of early mobilization and oral nutrition, and continuous monitoring and documentation of protocol compliance. They lead data collection and audit processes, organize regular interprofessional case discussions to provide structured audit and feedback, deliver ongoing training to clinical staff, and translate current evidence into nursing practice. At our clinic, ERAS staff also apply principles of implementation science by systematically evaluating adherence and developing targeted strategies to strengthen compliance where needed.

### Statistics

Descriptive statistics and data analysis were carried out using IBM SPSS Statistics (Version 30.0.10) and GraphPad Prism (Version 10.4.2). Descriptive statistics are presented as mean ± standard deviation (SD) or number (n) and percentage (%). For metric variables Welch t-test, Mann-Whitney U test and for nominal variables Chi2-test or exact Fisher-test were performed. The significance level (α-level) chosen was < 0.05. Both the log-rank test and the Cox proportional hazards model were applied to evaluate survival differences between groups. The log-rank test was used for a non-parametric comparison of Kaplan–Meier survival curves, while the Cox regression model was used to estimate the hazard ratio (HR) and its confidence interval.

## Results

### Patients characteristics

The study included 168 patients, 74 of whom were in the control group (Table 1**)**. These Non-ERAS control patients underwent liver resection according to the standard institutional protocol prior to the implementation of the ERAS program. The ERAS group comprised 94 patients. For further analysis, the ERAS group was subdivided into two cohorts: patients who were treated according to the ERAS program under the supervision of dedicated ERAS staff (*n* = 64), and those who received the ERAS protocol without ERAS staff supervision (*n* = 30).

**Table 1:** Patient characteristics of the Non-ERAS group (2014–2018) and ERAS group (2019–2024). The data are presented as mean ± SD or n (%) or median (IQR)

Parameter	Non-ERAS (*n* = 74)	ERAS (*n* = 94)	*p*
Age	65.4 ± 11.6	66.1 ± 11.0	0.696
Sex			
• female • male	31 (41.9) 43 (58,1)	39 (41.5) 55 (58.5)	1.000 1.000
BMI	25.5 ± 3.8	24.6 ± 4	0.417
ASA			0.050
• 1–2 • 3–4	46 (62.2) 28 (37.8)	43 (45.7) 51 (54.5)	
Diabetes mellitus	14 (18.9)	11 (11.7)	0.277
Klatskin type			0.337
• I • II • IIIa • IIIb • IV	4 (5.4) 3 (4.1) 25 (33.8) 14 (18.9) 28 (37.8)	9 (9.6) 2 (2.1) 42 (44.7) 11 (11.7) 30 (31.9)	
Preoperative stenting	64 (86.5)	84 (89.4)	0.568
Preoperative cholangitis	29 (39.2)	29 (30.9)	0.280
Adjuvant Therapy			0.002
• Yes • No • Unknwon	23 (31.8) 42 (56.8) 9 (12.2)	38 (40.2) 29 (30.9) 27 (28.7)	
Neoadjuvant Therapy	4 (5.4)	9 (9.6)	0.398
Resection status			0.056
• R0 • R1 • Unknown	55 (74.3) 19 (25.7) 0	64 (68.1) 23 (24.5) 7 (7.4)	
Lymph node status (+)			0.824
• Yes • No • Unknown	34 (46) 39 (52.7) 1 (1.4)	39 (41.5) 54 (57.4) 1 (1.1)	
Type of operation			0.050
• Right Trisectionectomy • Left Trisectionectomy • Extended right Hemihepatectomy • Extended left Hemihepatectomy • Right Hemihepatectomy • Left Hemihepatectomy • Bile duct resection only	41 (55.4) 1 (1.4) 7 (9.5) 24 (32.4) 0 (0) 1 (1.4) 0 (0)	48 (51.1) 7 (7.5) 10 (10.6) 21 (22.3) 3 (3.2) 0 (0) 5 (5.3)	
Length of stay (after surgery)			
Mean Median	31.3 ± 44.8 22.5 (14–31)	28.0 ± 24.5 20 (14–30)	0.320 0.180
Preoperative white blood cell count	7.99 ± 2.63	7.98 ± 2.43	0.987
Preoperative INR	1.05 ± 0.14	1.01 ± 0.12	0.343
Preoperative creatinine	0.90 ± 0.44	0.91 ± 0.41	0.767
Preoperative total bilirubin	1.57 ± 2.29	1.36 ± 1.51	0.512

No significant differences were observed between ERAS and Non-ERAS groups in terms of age, sex, BMI, diabetes mellitus, preoperative stenting, preoperative cholangitis, or neoadjuvant therapies. Also no significant differences in laboratory values were observed between the groups. Distribution of ASA scores was noted between groups, with more ASA 1–2 in the Non-ERAS group (Non-ERAS 62.2% vs. ERAS 45.7%) whereas more ASA 3–4 was noted in the ERAS cohort (Non-ERAS 37.8% vs. ERAS 54.5%), *p* = 0.050. With regard to the type of surgery Right trisectionectomy was the most commonly performed operation (Non-ERAS: 55.4%; ERAS: 51.1%), followed by extended left hemihepatectomy (Non-ERAS: 32.4%; ERAS: 22.3%) and extended right hemihepatectomy (Non-ERAS: 9.5%; ERAS: 10.6%). A trend toward a differential distribution of surgical approaches between the groups was observed. The distribution of Klatskin tumor types showed a trend between the groups. In the Non-ERAS cohort, Klatskin type IV was most frequent (37.8%), followed by type IIIa (33.8%) and type IIIb (18.9%). In contrast, the ERAS group showed a predominance of type IIIa (44.7%), followed by type IV (31.9%) and type IIIb (11.7%). However, these differences were not statistically significant. There was no statistically significant difference in length of stay (LOS) between groups, resection margin and lymph node status between both groups. The distribution of adjuvant therapy differed significantly between the groups (*p* = 0.002), with a higher proportion of patients receiving adjuvant treatment in the ERAS group. Notably, the proportion of cases with unknown adjuvant therapy status was also higher in this group.

### Overall adherence

Table 2 shows the different distribution of the different categories and subcategories of the ERAS protocol (see also Supplement 1 and 2). Total compliance was 64.3%, when only calculating perioperative items even 67.1%. Pre-admission adherence was the lowest at 56.2%, with high compliance in nutritional status assessment (96.9%) and patient education (92.2%), but no adherence to preoperative nutritional treatment (0%), low rates for smoking cessation (13.3%) and alcohol cessation due to limited time prior to surgery. Preoperative adherence showed the highest adherence rate with 80.7%, notably due to full compliance with antibiotic prophylaxis (100%) and good adherence to steroid administration (84.2%). However, thrombosis prophylaxis (65.1%) and sedative medication (74.6%) showed lower rates. Intraoperative adherence was low at 57.6%. For avoiding hypothermia and 0.9% NaCl full adherence was noted. However, avoidance of prophylactic abdominal drainage (7.1%) and nasogastric tubes (6.2%) showed particularly low compliance due to hepatobiliary reconstruction. Postoperative adherence was 62.9%, with significant variation. Mobilization on the day of surgery was low but improved markedly from postoperative day 1 onward, increasing adherence to 75.9% on POD1, 85.2% on POD2, and 94.5% on POD3.

**Table 2:** Different items of the ERAS protocol and grade of adherence; Results were calculated based on recorded compliance and non-compliance. Missing or inapplicable data were not included in the calculation. For some items (e.g., smoking cessation, alcohol withdrawal, nutritional therapy), the assessment is of limited use, as the underlying oncological disease and the need for liver resection as soon as possible meant that the required time component of, for example, 4 weeks could not be guaranteed in these cases. Note, items that were only added since the last guideline update 2022 were evaluated from this point onwards

ERAS Item	Compliance %	ERAS Item	Compliance %
**Pre-admission**	**56.2%**	**Intraoperatively**	**57.6%**
*Preoperative Nutritional Status Assessment*	*96.8*	*Skin preparation used*	*93.8*
*Preoperative Nutritional Treatment*	*0*	*Avoiding hypothermia*	*100*
*Smoking cessation*	*13.3*	*Regional Analgesia for Open Surgery*	*35.7*
*Alcohol cessation*	*50*	*Use of Omentum Flap For Left-sided Liver*	*60*
*Preadmission Patient Education*	*92.2*	*Use of 0.9% NaCl*	*100*
*Preoperative biliary drainage*	*84.6*	*Prophylactic abdominal drainage*	*7.1*
		*Avoidance of Nasogastric Tube*	*6.3*
**Preoperatively**	**80.7%**	**Postoperatively**	**62.9%**
*Preoperative Oral Carbohydrate Treatment*	*80.3*	*Postoperative glycaemic control*	*80*
*Thrombosis Prophylaxis*	*65.1*	*Duration of IV Fluid Infusion (nights)*	*17.2*
*Preoperative Sedative Medication*	*74.6*	*Weight Change on POD1*	*44.4*
*PONV Prophylaxis Administered*	*79.7*	*Postoperative artificial nutrition and early oral intake*	*66.1*
*Preoperative Steroid Administration*	*84.2*	*Mobilisation at all on day of surgery*	*17.2*
*Antibiotic Prophylaxis before Incision*	*100*	*Mobilisation on POD 1*	*75.9*
		*Mobilisation on POD 2*	*84.9*
		*Mobilisation on POD 3*	*94.4*
		*30 day follow up performed*	*85.7*
**Total**			
**Compliance incl preadmission**	**64.3**		
**Perioperative compliance**	**67.1**		

### Complications

Postoperative complication rates were compared between the Non-ERAS and ERAS cohorts (Table 3) and stratified by the Clavien–Dindo classification. For minor complications (Clavien–Dindo grades I–II), a significant reduction was observed in the ERAS subgroup with dedicated ERAS staff compared with the Non-ERAS group (14.1% vs. 28.4%; *p* = 0.042), whereas no significant difference was found when comparing Non-ERAS with the overall ERAS cohort (*p* = 0.051) or the ERAS subgroup without ERAS staff (*p* = 0.377). For major complications (Clavien–Dindo grades III–V), there were no statistically significant differences between groups. The overall complication rate (Clavien–Dindo grades I–V) was significantly lower in all ERAS groups compared with Non-ERAS (overall ERAS: *p* = 0.003; ERAS with staff: *p* = 0.007; ERAS without staff: *p* = 0.007).

**Table 3:** Complication for the different cohort compared to each other, Complication grade in Clavien-Dindo (CD) for all ERAS patients (*n* = 94), ERAS with ERAS staff (*n* = 64) and ERAS *without* ERAS staff (*n* = 30) each compared to the Non-ERAS group (*n* = 74)

Category	Complication rate % (*n*)		*p* value	Complication rate % (*n*)		*p* value	Complication rate % (*n*)		*p* value
	ERAS all	Non-ERAS		ERAS (with staff)	Non-ERAS		ERAS (no staff)	Non-ERAS	
**CD 1–2**	16 (15)	28.4 (21)	0.051	14.1 (9)	28.4 (21)	***0.042***	20 (6)	28.4 (21)	0.377
**CD 3–5**	67 (63)	68.9 (51)	0.974	70.3 (45)	68.9 (51)	0.859	60 (18)	68.9 (51)	0.823
**CD 1–5**	83 (78)	97.3 (72)	***0.003***	84.4 (54)	97.3 (72)	***0.007***	80 (24)	97.3 (72)	***0.007***

As shown in Table 4, patients with < 50% adherence to ERAS protocols had a significantly higher overall complication rate (Clavien-Dindo I–V) compared to those with ≥ 50% adherence (100% vs. 78.2%, *p* = 0.031). However, no statistically significant differences were observed in rates of minor (Clavien-Dindo I–II) or major complications (Clavien-Dindo III–V) across different adherence thresholds (≥ 50%, ≥ 60%, or ≥ 70%). While a trend toward fewer complications was noted with increasing adherence, these did not reach statistical significance beyond the 50% cut-off.

**Table 4:** Complication rates (Clavien-Dindo classification, CD) of the full ERAS cohort (*n* = 94) according to different adherence thresholds (Adh) (≥ 50%, ≥ 60%, ≥ 70%). Complications rate, n (%)

	Adh < 50% (*n* = 18)	Adh ≥ 50% (*n* = 46)	*p*	Adh < 60% (*n* = 34)	Adh ≥ 60% (*n* = 30)	*p*	Adh < 70% (*n* = 54)	Adh ≥ 70% (*n* = 10)	*p*
**CD 1–2**	4 (22.2)	5 (10.9)	0.255	5 (14.7)	5 (16.7)	1.000	8 (14.8)	1 (10)	1.000
**CD 3–5**	14 (77.8)	31 (67.4)	0.414	26 (76.5)	19 (63.3)	0.251	39 (72.2)	6 (60)	0.466
**CD 1–5**	18 (100)	36 (78.2)	**0.031**	31 (91.2)	23 (76.7)	0.169	47 (87)	7 (70)	0.182

### Survival analysis

**Fig. 1:**
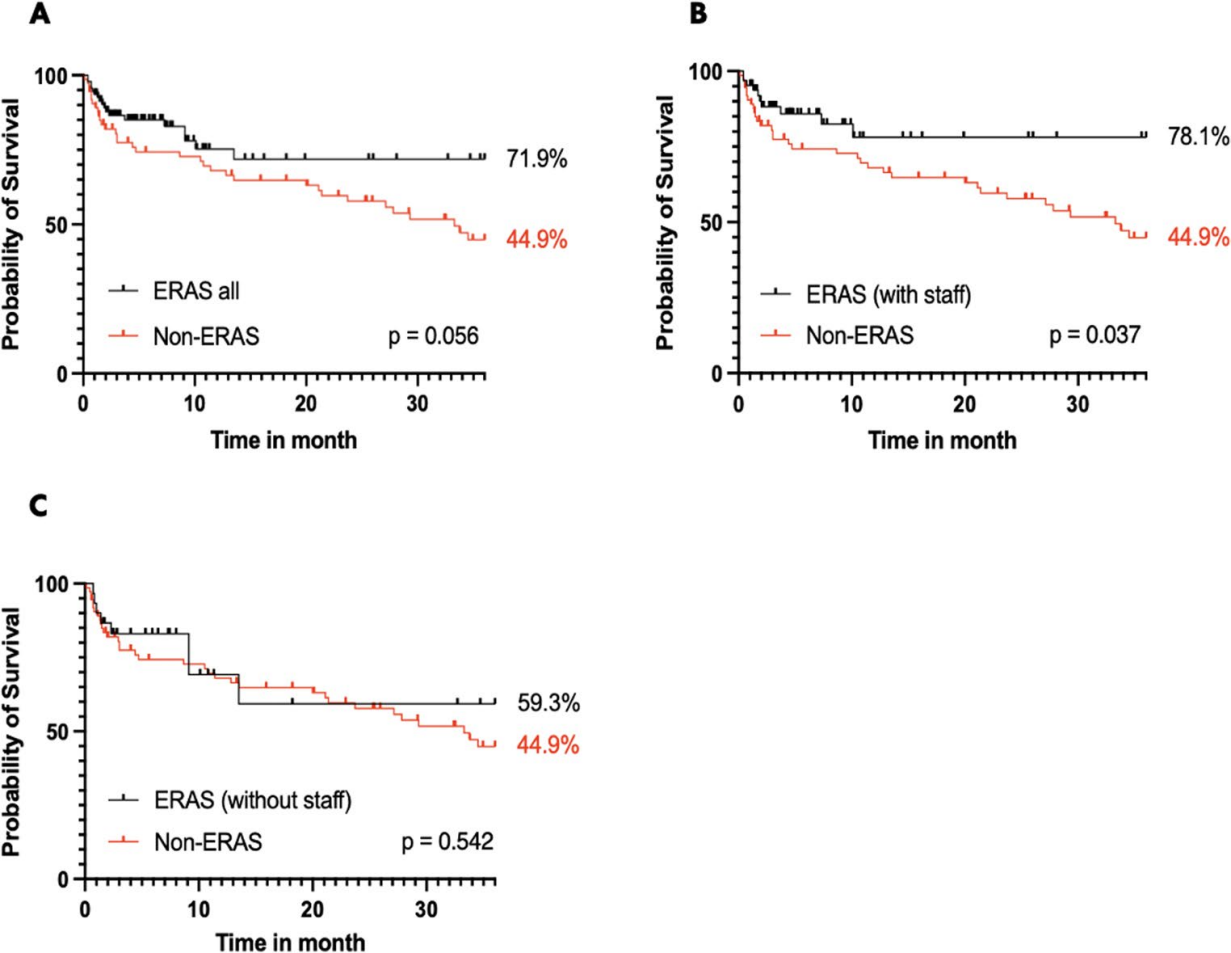
**(A)** Survival analysis between the ERAS group (ERAS all, *n* = 94) and the Non-ERAS group (*n* = 74), *p* = 0.057. HR = 0.572, *p* = 0.061. **(B)** Survival analysis between the ERAS group (with staff, *n* = 64) and Non-ERAS group (*n* = 74), *p* = 0.039. HR = 0.478, *p* = 0.044. **(C)** Survival analysis between the ERAS group (without staff, *n* = 30) and Non-ERAS group (*n* = 74), *p* = 0.535. HR = 0.783, *p* = 0.538. The log-rank test was used for statistics

Next, we analyzed survival (Fig. 1). The ERAS group showed a consistently higher survival probability over the 36-month follow-up than the Non-ERAS cohort. Although the difference did not meet the conventional threshold for statistical significance (*p* = 0.057; log-rank test), the data indicate a trend toward improved survival with ERAS-based perioperative care. This is further supported by a Cox proportional hazards analysis, which yielded a hazard ratio of 0.572 (*p* = 0.061), corresponding to an approximately 43% relative reduction in the hazard of death for patients managed within the ERAS framework. Next, we analyzed only ERAS patients who were supervised by dedicated ERAS staff. The ERAS group with supervision showed significantly improved survival (*p* = 0.049), with 78.1% alive in the ERAS group and 45.5% in the Non-ERAS group. This was also supported by Cox regression analysis, which demonstrated that ERAS implementation was associated with a significantly reduced hazard of death (HR = 0.478; *p* = 0.044). By contrast, among patients treated under ERAS protocols without dedicated ERAS staff support and those managed prior to ERAS implementation (Non-ERAS), there was no difference in survival (log-rank test: *p* = 0.535; HR = 0.783; *p* = 0.538).

A multivariable Cox proportional hazards regression analysis (Table 5) was subsequently performed to evaluate the impact of the ERAS program, oncological factors, and patient-related parameters on overall survival. Only younger age (≤ 65 years; *p* = 0.013, HR 0.446, 95% CI 0.235–0.846) and the administration of adjuvant chemotherapy (*p* = 0.012, HR 0.737, 95% CI 0.580–0.936) were identified as independent positive prognostic factors. Although ERAS implementation showed a trend towards improved survival, this effect did not reach statistical significance after multivariable adjustment.

**Table 5:** Univariate and multivariable cox regression analysis of factors associated with overall survival

Variable	Univariate analysis		Multivariate analysis	
	Hazard Ratio (95% CI)	*p*-value	Hazard Ratio (95% CI)	*p*-value
ERAS	0.548 (0.306–0.980)	**0.043**	0.628 (0.273–1.442)	0.272
ERAS (with staff)	0.488 (0.244–0.979)	**0.043**	0.707 (0.274–1.824)	0.473
Age ≤ 65	0.509 (0.285–0.911)	**0.023**	0.446 (0.235–0.846)	**0.013**
ASA ≤ 2	0.711 (0.412–1.227)	0.221	0.623 (0.347–1.120)	0.114
Neoadjuvant Chemotherapy	1.146 (0.355–3.702)	0.820	2.460 (0.709–8.536)	0.156
Clavien Dindo I-II	0.566 (0.266–1.204)	0.140	1.227 (0.243–6.202)	0.805
Clavien Dindo III-V	2.051 (1.029–4.089)	**0.041**	2.291 (0.534–9.836)	0.265
Adjuvant Chemotherapy	0.711 (0.540–0.938)	**0.016**	0.737 (0.580–0.936)	**0.012**
Resection margin (R0)	0.961 (0.803–1.51)	0.664	1.058 (0.864–1.297)	0.585
Lymph node status (+)	0.985 (0.767–1.264)	0.903	0.912 (0.676–1.231)	0.549
Preoperative cholangitis	0.820 (0.458–1.465)	0.502	0.860 (0.466–1.589)	0.631

## Discussion

This study aimed to assess the impact of ERAS implementation in patients with PHC undergoing liver resection. Adherence to ERAS Society guidelines in the context of extended hepatic resections was associated with a marked decrease in postoperative complications and an improvement in long-term survival. These effects were most notable when the ERAS protocol was actively coordinated and overseen by dedicated ERAS staff.

There were no significant differences observed for baseline characteristics in our cohort except for ASA score, with ASA 1–2 being more representative in the Non-ERAS group, while ASA 3–4 occurred more often in the ERAS group and the administration of adjuvant chemotherapy, which is probably because of the clinical implementation of the recommendation of adjuvant chemotherapy after the results of the BILCAP trial became evident [14]. Distribution of age and sex were similar to other studies on use of ERAS in liver surgery [13, 15]. Distributions of surgical approach are similar to other studies. In our cohort, right or extended right hemihepatectomy and right trisectionectomy were carried out in 64% of the cases (both ERAS and Non-ERAS), comparable to 52% in a benchmark study across 24 high-volume centers [8]. LOS was numerically shorter in the non-ERAS cohort; however, this difference did not reach statistical significance for either mean (*p* = 0.320) or median LOS (*p* = 0.180). Importantly, this finding remained unchanged after exclusion of extreme outliers (LOS > 100 days). Benchmark data from Mueller et al. report a median LOS of 23 days (range 3–213) following resection for PCH [8]. In this context, the LOS observed in both groups of the present study falls well within previously reported ranges, supporting the external validity of our cohort. Although LOS is frequently used as an outcome parameter in the evaluation of ERAS programs, it should not be interpreted as a standalone surrogate for surgical quality. LOS is influenced by multiple factors beyond postoperative morbidity, including discharge logistics, institutional practices, and patient-specific recovery patterns.

Adherence rate for the use of the ERAS protocol for PHC is 64.3% in our cohort. After implementation of the ERAS program for liver surgery in our clinic adherence was 62,5% in the first year 62,5% but increased over time to even 72,5% for all liver tumors during study time [11, 12]. Evaluations of adherence in patients with PHC are rare. A study conducted by Jongkatkorn et al. also showed very low adherence to the ERAS protocol for cholangiocarcinoma, lower than in our cohort, while they used only 17 items from the ERAS guideline [16]. The study included both PHC and intrahepatic cholangiocarcinoma, which do not always require complex reconstruction and can often be resected minimally invasively. For PCH only 14,2% achieved adherence rates above 50% The question rising here is why is the adherence rate for PCH lower than for other liver surgeries. Patients with PHC often require extended liver resection with hepatobiliary reconstruction that goes along with the high risk of complication rates. That surgical complexity and severity can affect adherence has been shown previously by our group in a study of 243 patients undergoing simple and complex minimally invasive liver resections [17] Adherence to ERAS measures decreased with higher complexity (*P* < 0.001). Indeed, patients who experience complications may be unable to perform some of the required items. Moreover, the extent of the surgery and the open access approach, for instance, can also result in reduced mobility post-operatively. Additionally, some items in the ERAS guidelines, such as the omission of abdominal drains and nasogastric tubes, may not be applicable when a hepaticojejunostomy is performed. In this particular context, it may be advisable to adapt adherence and ERAS items of the guidelines.

Complication rates for PHC remain high. Benchmark values from our cohort are comparable to those of other centers for PCH. Clavien-Dindo grade ≥3a complications ≤ 70% was defined as the 75th percentile benchmark value by Mueller et al., which is in line to our cohort [8]. Note that a 3-month follow-up period was used for postoperative morbidity. Quinn et al. also reported an overall complication rate of 87% for their ERAS cohort [13]. When it comes to the impact of the ERAS program on complication rates in PHC, no other study was found that systematically evaluated the rate of complications between Non-ERAS and ERAS groups. However, our study is in line with studies on ERAS in liver surgery that have shown reduced complications [11, 12, 18, 19]. In our study, the overall decrease in complications in the ERAS cohort regardless of ERAS staff supervision compared to the Non-ERAS group was mostly driven by a reduction in minor complications (ERAS all 16% vs. Non-ERAS 28.4%, *p* = 0.051; ERAS (with staff) 14.1% vs. Non-ERAS 28.4%, *p* = 0.042). A similar trend was shown in a previous study regarding complications in liver surgery by our group. Here a significant decrease in general complications (*p* = 0.033), mostly driven by a reduction in infection related complications (*p* = 0.007), was observed in a cohort of 1049 patients. A reduction in surgery related complications was not noted [12]. A meta-analysis of 646 patients cirrhotic patients who underwent liver surgery by Delabays et al. similar trends were observed. For minor complications a significant reduction in the ERAS cohort was shown (*p* = 0.0005) whereas not significant differences occurred for major complications [19]. Schmelzle et al. also already presented data from our center on all liver tumors after the first year of implementation of the ERAS program underlining the trend that adherence to ERAS items may reduce complication rates. In the high adherence group total complication rate was 12% compared to 40.6% in the low adherence group, *p* < 0.001 [11].

In our cohort, we observed a significantly improved survival in the ERAS cohort that was supervised by ERAS staff compared to the Non-ERAS cohort (*p* = 0.037). An increase in survival was also observed for the full ERAS cohort compared to the Non-ERAS group. Although this did not reach statistical significance (*p* = 0.056), a trend was evident. Studies on hepatectomy in liver cancer have shown prolonged survival among ERAS patients; for example, Zhang et al. reported significantly higher overall survival for ERAS versus Non-ERAS patients in a cohort of 1,143 patients (1 year: 93.1% vs. 89.3%, *p* = 0.041; 3 years: 68.7% vs. 61.2%, *p* = 0.035) [20]. However, this study mixed hepatocellular carcinoma (80.6%), cholangiocarcinoma (9.4%) and mixed hepatocellular carcinoma and cholangiocarcinoma tumors (10%).

In the multivariable analysis, ERAS was not identified as an independent predictor of improved overall survival, although a non-significant trend was observed. Only younger age and the administration of adjuvant chemotherapy were significantly associated with prolonged survival. Importantly, the implementation of the ERAS program at our institution temporally coincided with the dissemination of the BILCAP trial results and the subsequent routine use of adjuvant capecitabine in patients with PCH. This overlap represents a potential source of bias and may confound the interpretation of a survival benefit attributable to ERAS. Nevertheless, ERAS reduces postoperative morbidity and facilitates recovery. In this context, ERAS may indirectly influence long-term outcomes by enabling earlier initiation and improved delivery of adjuvant chemotherapy.

It has been shown that adherence can improve over time when the individual components of the ERAS program are introduced and implemented consistently and when a well-staffed ERAS team is in place [12, 21]. The fact that adherence increases over time was already shown in a previous study by Oehring et al. Here adherence rates raised consecutively from 62.5 to 72.5% (2019–2023) [12].Another major issue is maintaining consistent ERAS staffing throughout the year, e.g. our team fluctuates between two and four people. This has an impact on our ERAS program. In our cohort, 68.1% of the 94 patients had complete ERAS documentation, while the remaining patients were not seen or supervised by our dedicated ERAS staff. This was due to the COVID-19 pandemic, occasional understaffing around holiday periods such as Christmas and New Year or patients’ refusal to conduct in the ERAS program.

### Limitations

The limitations of this study primarily stem from its retrospective character although the EIAS database is prospectively handled. Moreover, the observational design and the absence of randomization are further possible limitations. Especially the non-randomized pre/post ERAS design is inherently susceptible to temporal bias. As ERAS implementation was time-dependent, chronological bias cannot be fully excluded. Over the study period, improvements in surgical techniques, perioperative care pathways, anesthetic management, and institutional experience may have influenced outcomes independently of ERAS implementation. Due to the complete collinearity between treatment period and ERAS exposure, these effects could not be statistically disentangled. Furthermore, this is a single-center study with a relatively small sample size compared to other ERAS studies in liver surgery, which is attributable to the rarity of PCH. Although this may limit statistical power, to our knowledge, comparable cohorts specifically assessing ERAS in PCH have not been reported. ERAS inclusion was heterogeneous due to several reasons such as missing ERAS staff. Therefore potential changes in patient selection cannot be fully excluded. Although the study reflects real-world clinical practice, causality between ERAS implementation and improved outcomes cannot be definitively established.

## Conclusions

The implementation of an ERAS protocol in patients undergoing extended liver surgery for PCH, requiring biliary or even vascular reconstruction, was feasible and associated with improved perioperative recovery. Postoperative morbidity was reduced, mainly due to a lower rate of minor complications. Although there were indications of improved survival in the ERAS group, this observation should be interpreted cautiously given the observational design of the study and the potential for bias. Nevertheless, by reducing postoperative morbidity and facilitating recovery, ERAS may indirectly improve long-term outcomes through earlier initiation of adjuvant chemotherapy.

Adherence to individual ERAS elements remained limited, partly because several items are not fully applicable in patients undergoing surgery for PHC, while others require thresholds that are difficult to achieve in this population. In our view, these findings highlight the importance of dedicated ERAS-trained staff for successful protocol implementation. Although the present study provides important preliminary insights, a randomized controlled trial is required to strengthen the evidence base and confirm these findings.

## Data Availability

The datasets generated during and analysed during the current study are available from the corresponding author on reasonable request.

## Declarations

### Declarations of AI and AI-assisted technologies in the writing process

During the preparation of this work the author(s) used ChatGPT 5.4 in order to maintain clarity, correct grammatical errors and to visualize conceptual content. After using this tool/service, the author(s) reviewed and edited the content as needed and take full responsibility for the content of the publication.
